# Anti-NMDA Receptor Encephalitis Associated with Ovarian Teratoma: A Comprehensive Review

**DOI:** 10.3390/biomedicines14092040

**Published:** 2026-09-10

**Authors:** Hanna Miski, Kamila Krupa, Bartosz Balasiewicz, Dominik Kucharski, Hubert Rzewuski, Andrzej Deptała, Anna Badowska-Kozakiewicz

**Affiliations:** 1 Students’ Scientific Organization of Cancer Cell Biology, Department of Oncology Propaedeutics, Medical University of Warsaw, 01-445 Warsaw, Poland; s090695@student.wum.edu.pl (H.M.); kamila.krupa@student.wum.edu.pl (K.K.); s090212@student.wum.edu.pl (B.B.); s090400@student.wum.edu.pl (D.K.); s090538@student.wum.edu.pl (H.R.); 2 Department of Oncology Propaedeutics, Medical University of Warsaw, 01-445 Warsaw, Poland; andrzej.deptala@wum.edu.pl

**Keywords:** anti-N-methyl-D-aspartate receptor encephalitis, ovarian teratoma, autoimmune encephalitis, tumor-associated autoimmunity, neuroimmunology, B-cell-mediated immunity, GluN1 subunit, paraneoplastic neurological syndromes

## Abstract

Anti-N-methyl-D-aspartate receptor encephalitis (anti-NMDAR-E) is a severe autoimmune encephalitis that predominantly affects young women and is frequently associated with ovarian teratomas. Increasing evidence indicates that these tumors play a central role in disease initiation by providing a peripheral immunological trigger. This comprehensive review summarizes current knowledge on the immunopathogenesis, clinical manifestations, diagnostic approach, therapeutic strategies, and long-term outcomes of ovarian teratoma-associated anti-NMDAR-E. We discuss the unique immunogenic properties of ovarian teratomas, including the presence of neuroglial tissue expressing the GluN1 receptor subunit (formerly known as NR1) of the N-methyl-D-aspartate receptor (NMDAR), dense immune cell infiltrates, and tertiary lymphoid structure-like formations that support local antibody production. These features are thought to promote molecular mimicry and loss of immune tolerance, leading to B-cell activation, intrathecal antibody synthesis, and antibody-mediated neuronal dysfunction. Furthermore, we review the characteristic multistage clinical course, key diagnostic findings in cerebrospinal fluid, imaging and electrophysiology, and the importance of early tumor removal combined with immunotherapy. Emerging therapeutic approaches and experimental models that provide insight into disease mechanisms and novel treatment targets are also highlighted. An improved understanding of tumor-driven neuroimmunological interactions is essential for earlier diagnosis, optimized treatment strategies, and better long-term outcomes in affected patients.

## 1. Introduction

Anti-NMDAR-E is an autoimmune disease associated with the presence of antibodies against the GluN1 subunit in the CSF, defined by significant neuropsychiatric symptoms [1,2]. Anti-NMDAR-E is considered the most common form of autoimmune encephalitis and is increasingly recognized in clinical practice; however, its true incidence remains uncertain [3]. The most relevant triggers of the disease include ovarian teratomas and herpes simplex virus 1 (HSV-1) encephalitis; nonetheless, the majority of cases are idiopathic [4,5,6]. Anti-NMDAR-E can occur across a broad range of age groups; however, it typically involves young adults, predominantly females, although the disease can affect males [1,5,7]. Warren et al., in the review of demographic data from 633 patients, reported that the mean age of onset was 22.6 years (age range from 8 months to 84 years), although 253 cases were below 18 years. Among these patients, 492 (77.8%) were females and 141 (22.2%) were males. Furthermore, viral prodrome was observed in 133 cases (21.0%) with symptoms of fever and headache at the time of onset. Ovarian teratoma was identified in 178 cases (28.1%), and other malignant neoplasms were identified in 28 cases (4.4%) [8]. According to Titulaer et al., the proportion of male patients is higher in the age group above 45 years and among prepubertal patients, which may indicate the role of hormonal factors in disease development [9].

Ovarian teratomas are the most common tumors belonging to the ovarian germ cell tumor (OGCT) family [10]. Teratomas originate from totipotent or primitive germ cells and are formed by somatic-type tissues (atypical to anatomic location of the tumor) derived from cells belonging to more than one of the primitive embryonic (ectodermal, mesodermal, endodermal) layers [11,12,13]. Teratomas may be classified as mature (solid or cystic benign neoplasms), immature (malignant), or monodermal (specialized) [11,12].

Mature cystic teratomas (MCTs), also referred to as dermoid cysts, are the most common benign OGCTs [14,15]. As reported by Westhoff et al., the incidence of MCTs is 8.9 cases per 100,000 women [16]. MCTs typically develop in young women at their reproductive age, although they may occur in women of any age [17]. A large retrospective study, including 501 cases, claims that patients’ median age was 30 years, while the age ranged from 13 to 76 [18]. The risk factors associated with the occurrence of MCTs are alcohol use, late menarche with irregular menstruation, infertility, a low number of pregnancies, cystic teratoma in one’s medical history, and physical activity during adolescence (related to an anovulatory menstrual cycle) [16]. According to another study of 305 ovarian teratomas, most MCTs are between 5 and 9 cm in size (average being 7.7 cm), while the largest lesion was 38 cm [19]. MCTs are more commonly located on the right ovary (72%), although in about 10% of cases they can be found bilaterally [20].

The cystic cavity of MCTs is lined with a squamous epithelium and typically filled with sebaceous material that is liquid at body temperature [21]. The cyst is mostly unilocular, and a dermal papilla (called Rokitansky tubercle) can usually be found extending into the lumen, which is an important ultrasonographic finding in MCT diagnosis [22,23,24]. Structures like bones, teeth, and hair are typically found within this protuberance [24]. Considering ectodermal derivatives, Ayhan et al. claims that skin and skin appendages were found in 100% of samples, and neural tissue was found in 82.5% of cases [18].

Immature teratomas (ITs) occur with an estimated prevalence of 1.5 per 100,000 women [25]. According to Smith et al., ITs constituted 35.6% of all malignant germ cell tumors [26]. Moreover, the highest incidence of ITs is in the first two decades of life [27]. Upon gross examination, the tumors were typically unilateral and larger than mature cystic teratomas. Histologically, immature teratomas contain both mature and immature tissues derived from embryonic germ cell layers, where immature neuroepithelium is the most significant diagnostic and grading feature [28].

Patients who had anti-NMDAR-E associated with ovarian teratoma were considerably younger, with a mean age of 20.8 years compared to 42.3 years in patients without anti-NMDAR-E. Ovarian teratomas in patients with anti-NMDAR-E were typically unilateral and smaller (with a mean maximum diameter of 26.1 mm) than in non-anti-NMDAR-E patients (67.0 mm). Moreover, anti-NMDAR-E-associated teratomas had a lower frequency of teeth or calcifications and had lower rate of occupation by fat components [29]. Of note, another study shows that immature teratomas are more common in anti-NMDAR-E patients (11.8% of cases) than in patients without anti-NMDAR-E (3% of cases) [30].

## 2. Immunogenic Properties of Ovarian Teratoma in Anti-NMDAR-E

### 2.1. Presence and Characteristics of Neural Tissue in Teratomas

Chefdeville et al. reported that the immune environment of neuroglial tissue in ovarian teratoma is associated with autoimmunity directed against the onconeural antigen NMDAR. In this study, nervous tissue was identified in all except one (26/27, 96%) of ovarian teratomas associated with anti-NMDAR-E. In comparison, only about one third of control (non-anti-NMDAR-E associated) teratomas contained neural tissue [31]. Day et al. suggest that abnormal neurons found in neuroglial tissue distinguish ovarian teratomas associated with anti-NMDAR-E and promote the development of autoimmunity [32]. Moreover, these neuroglial components demonstrated increased glial cellularity and elevated glial marker expression and are distinguishable from reactive abnormalities commonly found in ovarian teratomas [31,32].

### 2.2. Expression of GluN1, Antigenic Mimicry, and Immune Infiltration

Furthermore, these abnormal neurons express the GluN1 subunit of the NMDAR, confirming that the same antigen targeted by patients’ antibodies in the CNS is present within the tumor itself and thereby providing a direct antigenic source for autoimmunity [33,34]. In addition to these tumor-specific features, some studies identified dense immune infiltrates adjacent to the neuroglial tissue [31,32,33,35,36,37]. These lymphoid infiltrates are formed by B-cells, T-cells, and dendritic cells. Moreover, they may organize tertiary lymphoid structures with IgG and IgA deposits in direct contact with the neural tissue [31,36]. Additionally, the deposition of complement component C3d around neuroglial regions suggests active intratumoral humoral immunity. The neuroglial component of teratomas expresses genes characteristic of CNS tissue, including GRIN1, which encodes GluN1, glial markers, synaptic proteins, and the up-regulation of inflammatory cytokine transcripts such as interleukin-6 (IL-6) and TNF-α. These neural components replicate the architecture of the central nervous system, thereby creating an antigenic mimic of the brain tissue [38,39]. This molecular similarity gives rise to molecular mimicry, whereby lymphocytes activated against tumor antigens cross-react with endogenous neuronal receptors, thereby providing a mechanistic link between ovarian teratoma and autoimmune encephalitis [10].

### 2.3. Tertiary Lymphoid Structures and Local Antibody Production

The immune architecture of these tertiary lymphoid structure-like (TLS-like) structures includes follicular dendritic cells and class-switched plasma cells, suggesting that local B-cell activation and maturation occur within the tumor [31]. Makuch et al. observed that B lymphocytes, which are present in ovarian teratoma, can produce IgG against GluN1 in vitro [36]. Altogether, these findings support the concept that the teratoma functions as a peripheral site of immune initiation, providing both the antigenic target and the immunological machinery necessary to break tolerance [31,32,33,34,35,36,37,40]. Nonetheless, further studies are required to describe molecular triggers that lead to the loss of immune tolerance and induce autoimmune disease [31]. Despite the presence of neuroglial tissue and GluN1 expression in many teratomas, only a small subset of patients develop anti-NMDAR-E. Indeed, only 3.07% of 163 patients with confirmed ovarian teratoma were diagnosed with anti-NMDAR-E [41]. Several hypotheses have been proposed to explain this discrepancy. Although the presence of neuroglial tissue expressing GluN1 is thought to be an important contributor to disease development, it appears to be insufficient on its own to trigger anti-NMDAR-E, suggesting that additional tumor- and host-related factors may be involved [31,32]. As described in Section 2.1, Day et al. identified atypical (dysplastic) neurons in four of five teratomas from patients with anti-NMDAR-E, whereas such neurons were absent from all 39 control teratomas. In contrast, the presence of CNS neuronal tissue itself did not significantly differ between cases and controls (4/5 vs. 20/39; *p* = 0.36). Abnormal neuroglial elements were also associated with inflammatory infiltrates in anti-NMDAR-E-associated teratomas. These findings suggest that abnormalities of the neuronal component and their association with local inflammation, rather than the presence of neuroglial tissue alone, may contribute to the development of autoimmunity [32].

The presence of tertiary lymphoid structures and local GluN1-specific antibody production described above indicated that local immune processes may also be relevant [36]. However, whether differences in antigen presentation or local B-cell selection distinguish teratomas that trigger anti-NMDAR-E from those that do not has not been established.

The mechanisms explaining why some teratomas trigger a pathogenic immune response whereas others do not remain incompletely understood [31]. Furthermore, evidence supports antibody production both within ovarian teratomas and the CNS, indicating that peripheral and intrathecal immune responses may contribute to disease pathogenesis [36,39,42]. Genetic susceptibility represents a more speculative potential contributing factor. Although genetic associations have been reported, currently available evidence remains limited and genetic risk markers remain under investigation [43,44].

## 3. Immunopathogenesis of Anti-NMDAR-E

**Figure 1:**
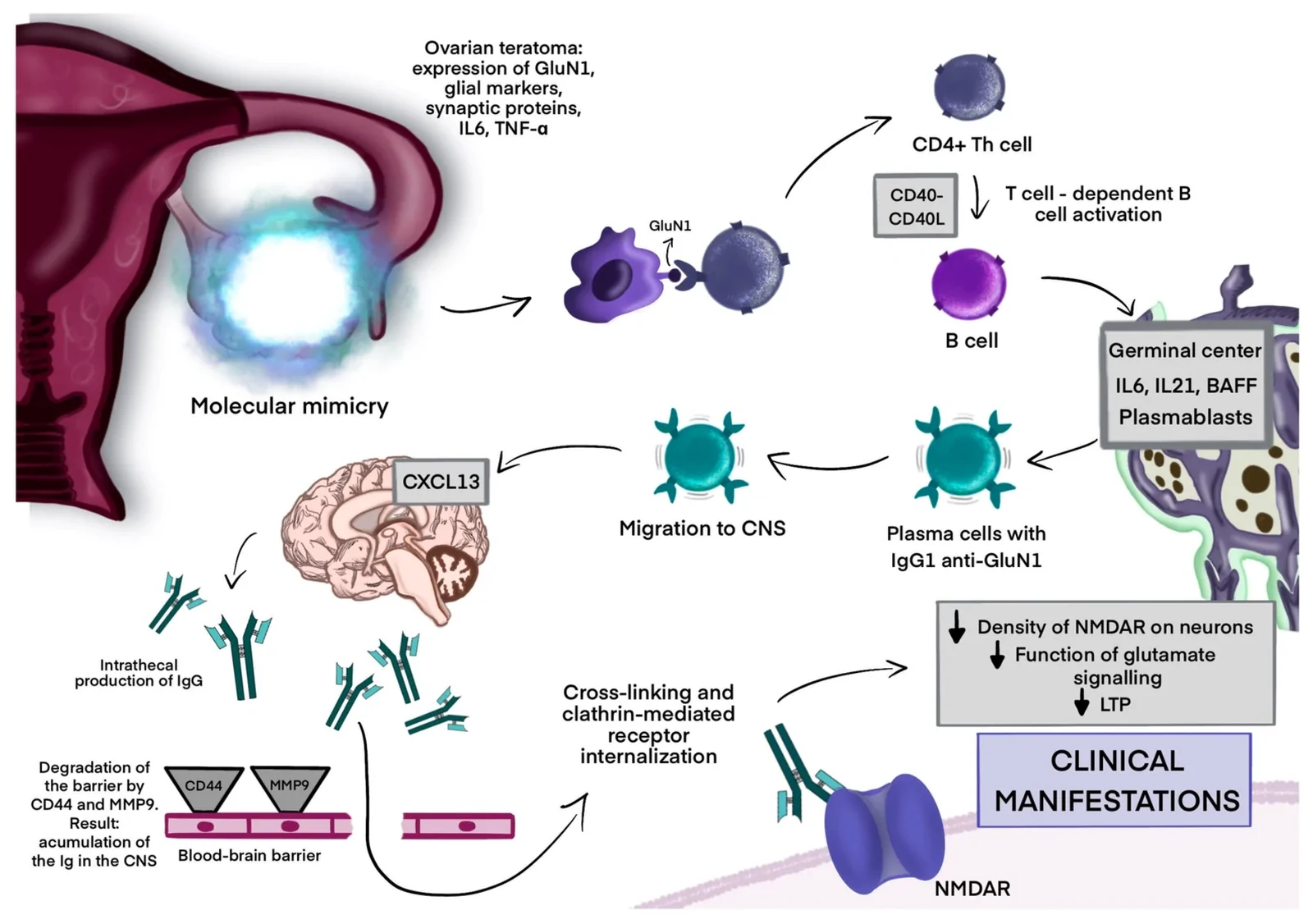
The immunopathogenesis of anti-NMDAR-E. Schematic overview of the immunopathogenic mechanisms underlying anti-NMDAR-E associated with ovarian teratoma. Tumor-derived GluN1 is presented by APC to CD4+ Th cells, driving T-cell-dependent B-cell activation and further IgG1 anti-GluN1 antibodies production, which migrate to the CNS. Intrathecal antibody production and binding of IgG to NMDARs cause cross-linking and receptor internalization, leading to anti-NMDAR-E. Based on Refs. [31,36,39,42,45,46,47,48,49,50,51,52,53,54,55,56]. Abbreviations: IL-6, interleukin 6; CD4+ Th cell, CD4+ T helper cell; APC, antigen-presenting cell; IL-21, interleukin 21; BAFF, B-cell-activating factor; BBB, blood–brain barrier; LTP, long-term potentiation; CNS, central nervous system. The arrow represents “reduced.”

Anti-NMDAR-E, caused by autoantibodies against the GluN1 subunit of NMDAR on the neuronal cell surface, is the most prevalent and severe type of autoimmune encephalitis [38]. As described in Section 2, anti-NMDAR-E associated with teratomas uniquely contains abundant neuroglial tissue. Tumor-derived GluN1 antigen acts as the peripheral immunological trigger (Figure 1) [31]. The activation of B lymphocytes begins in the peripheral immune environment. When antigen is presented by dendritic or stromal cells, T helper cell-dependent B-cell activation is initiated through MHC class II-peptide recognition and CD40-CD40L co-stimulation [45,46]. Cytokines, including IL-6, interleukin-21 (IL-21), and B-cell-activating factor (BAFF), enable activated B-cells to undergo somatic hypermutation and class-switch recombination within germinal-center-like structures. This process leads to development of class-switched, high-affinity plasma cells that secrete IgG1 antibodies that target the GluN1 subunit’s extracellular amino-terminal domain [47,48]. Elevated CXCL13 chemokine gradients in the CSF during the acute phase of the disease facilitate the migration of a subset of antibody-secreting plasmablasts into the CNS (Figure 1) [49].

Anti-NMDAR-E is characterized by strong B-cell and plasma-cell responses in both the peripheral and central compartments. Dense perivascular infiltrates of CD20-positive B-cells and CD138-positive plasma cells secreting immunoglobulin G (IgG) are detected in the brain tissue of affected patients, indicating intrathecal antibody production as opposed to passive diffusion from serum [42]. Moreover, Dalmau et al. found anti-GluN1 IgG antibodies in cerebrospinal fluid (CSF) of all studied patients, with CSF titers exceeding serum levels, proving local synthesis [39].

Under normal conditions, through endothelial tight-junction proteins, including occludin, ZO-1, and claudin-5, the blood–brain barrier (BBB) tightly controls the molecular exchange between the circulation and the CNS [51]. In the case of anti-NMDAR-E, pro-inflammatory cytokines and matrix-degrading enzymes temporarily disrupt this barrier, allowing circulating antibodies to enter the CNS. It has been observed that endothelial activation and junctional disruption, which increase paracellular permeability, are correlated with elevated levels of IL-6, IL-17, and TNF-α and related mediators in serum and CSF [48]. Moreover, the concurrent overexpression of CD44 and matrix metalloproteinase-9 (MMP-9) promotes leukocyte trafficking and basement membrane degradation, further destabilizing BBB integrity (Figure 1) [50].

In the CNS, plasmablasts maintain intrathecal antibody synthesis, as demonstrated by the presence of oligoclonal IgG bands and persistent CSF antibody production [54]. By binding to surface NMDARs, these antibodies induce receptor cross-linking and internalization (Figure 1) [52,57]. The bivalent structure of pathogenic anti-NMDAR antibodies enables them to cross-link adjacent NMDARs, thereby promoting receptor internalization. In contrast, monovalent antibody formats do not induce this cross-linking-dependent internalization, highlighting the importance of antibody valency in this mechanism [52,57]. This distinction provides the mechanistic rationale for ART5803, a monovalent antibody discussed in Section 6.5, which binds the GluN1 N-terminal domain and blocks pathogenic antibody binding without inducing receptor internalization [57]. This leads to reduced receptor density and a transient reduction in glutamatergic signaling, resulting in the disorder’s characteristic cognitive, behavioral, and motor symptoms (Figure 1). Furthermore, this process is reversible and non-cytotoxic. Receptor density and neuronal function return when antibody titers drop after immunotherapy or tumor removal [52,53].

This results in the rapid, reversible attenuation of NMDAR-mediated currents and impaired long-term potentiation (LTP), the cellular foundation of learning and memory [55]. Additionally, prolonged antibody exposure disrupts excitatory–inhibitory balance and large-scale network connectivity, resulting in the neurological manifestations characteristic of encephalitis, such as seizures and neuropsychiatric symptoms (Figure 1) [56].

## 4. Clinical Manifestations

### 4.1. Clinical Course and Prodromal Symptoms

**Figure 2:**
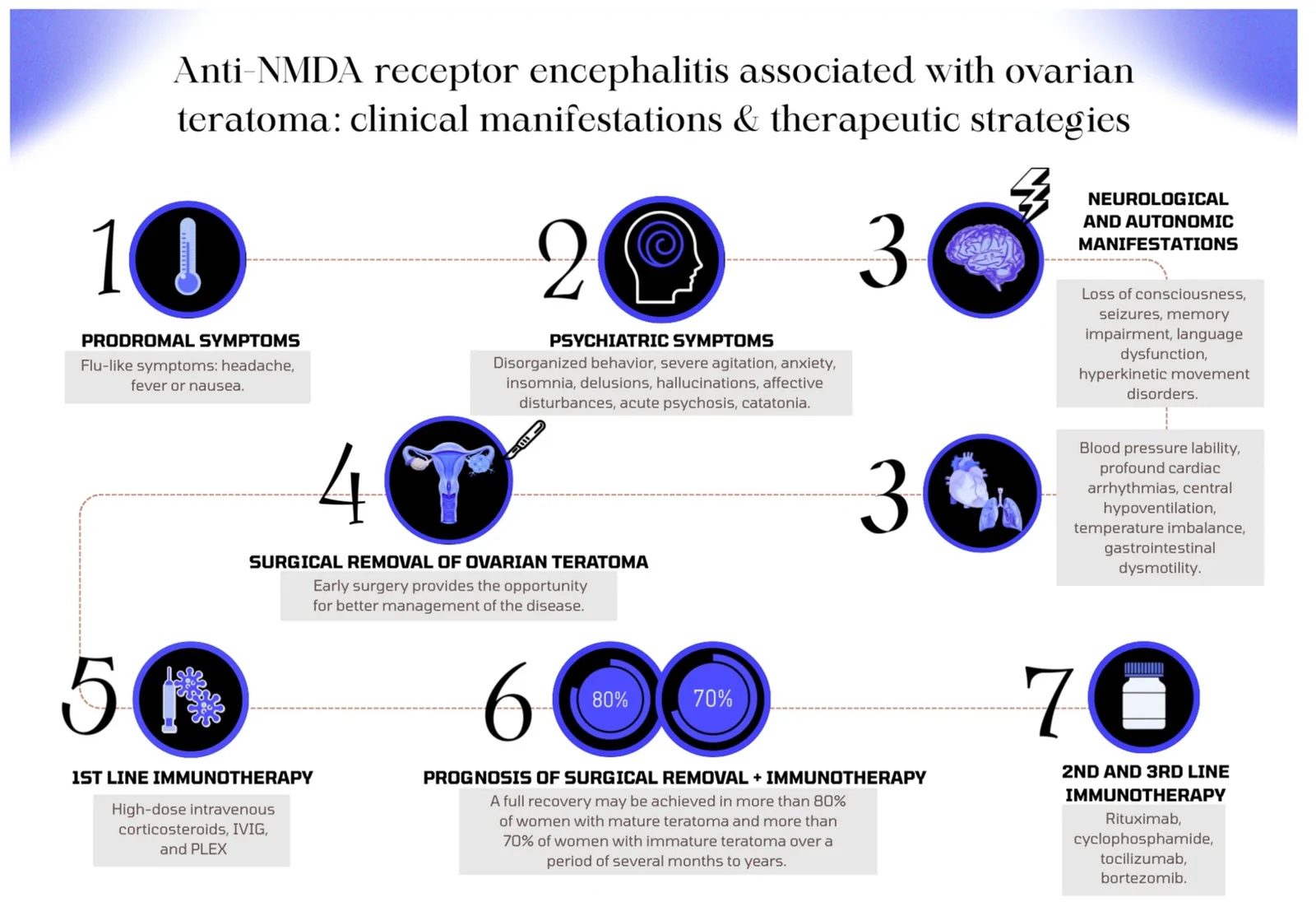
Clinical manifestation and therapeutic strategies. Overview of the clinical course and therapeutic management of anti-NMDAR-E encephalitis. The disease commonly progresses from prodromal symptoms to psychiatric, neurological, and autonomic manifestations. Treatment includes tumor resection when applicable, first-line immunotherapy, and escalation to second- or third-line immunosuppressive therapies in refractory cases. Based on Refs. [5,10,39,58,59,60,61,62,63,64,65,66,67].

The clinical course of anti-NMDAR-E typically follows a multistage progression, often beginning with prodromal flu-like-symptoms (headache, fever, or nausea) and a rapid onset of psychiatric disturbances (abnormal behavior or cognitive decline), followed by prominent neurological symptoms (seizures, memory and speech dysfunctions, abnormal movements), and potentially culminating in autonomic dysregulation (decreased level of consciousness and respiratory instability) [39,58,59]. Psychiatric symptoms commonly predominate early in adults, whereas children may present initially with seizures or movement disorders, as behavioral changes may be overlooked [59]. The transition from psychiatric to neurological and subsequently to the autonomic phase often occurs over days to weeks, which creates a temporal pattern that may help to distinguish anti-NMDAR-E from other psychiatric disorders (Figure 2) [59,60].

### 4.2. Psychiatric, Neurological, and Movement Disorders

Psychiatric symptoms are the most frequent presenting feature (60–80%) in adults and commonly include acute psychosis, severe agitation, anxiety, insomnia, delusions, hallucinations, and affective disturbances (Figure 2) [5,58,63,67]. Catatonia and disorganized behavior are also characteristic [58]. In a study by Wang et al., the majority of patients (95%) exhibited at least one of the psychiatric symptoms [64].

In a study by Dalmau et al., it was indicated that while psychiatric symptoms are predominant early, more than 95% of patients develop neurological symptoms eventually [39]. Neurological features of anti-NMDAR-E, as the disease advances, encompass loss of consciousness, seizures (including generalized or focal seizures), memory impairment (particularly episodic and anterograde amnesia), language dysfunction (dysphasia, mutism), and a spectrum of hyperkinetic movement disorders—chorea, orofacial dyskinesias and dystonia, stereotypies, myorhythmias, and abnormal postures (Figure 2) [5].

Cognitive deficits, short-term memory loss, and deficits in executive functions are often profound and may persist in many long-term survivors [10].

### 4.3. Autonomic Dysfunction

The autonomic stage may occur at various points during the clinical course, sometimes even throughout the disease, culminating and affecting the prognosis [10,61]. It usually manifests as profound cardiac arrhythmias, blood-pressure lability, central hypoventilation, and temperature dysregulation [5]. Consequently, dysautonic cardiorespiratory instability frequently drives admission to intensive care units and is associated with significant morbidity [5,61,62]. Specific complications such as sinus node dysfunction and sinus bradyarrhythmia requiring pacing have been reported [10]. Severe central hypoventilation often requires mechanical ventilation [65]. Temperature fluctuations, periods of hyperthermia or hypothermia, and diaphoresis may further complicate critical care management [10,66]. Gastrointestinal dysmotility and hypersalivation also contribute to the clinical picture (Figure 2) [61]. The risk of autonomic manifestations strictly correlates with severe encephalopathy and increases significantly when diagnosis and immunotherapy are delayed [5,63]. Close monitoring and multidisciplinary critical care interventions are therefore crucial components of management for patients who progress to this stage [5,63].

### 4.4. Misdiagnosis and Its Clinical Importance

Because the syndrome commonly affects young women, it can be initially misdiagnosed as a primary psychiatric disorder, leading to delays in appropriate neurological investigation [68,69]. Failure to recognize the staged presentation of the disease is a major contributor to diagnostic delay and poor clinical outcome [5]. Catatonia and disorganized behavior could be initially mistaken for schizophrenia [58,60]. Frequently, those symptoms prompt psychiatric referral before neurological signs emerge, resulting in the misdiagnosis of a previously healthy young woman, before autoimmune etiology is considered [5,58]. Importantly, specific histopathological and immunological features of ovarian teratomas may contribute to the pathogenesis of anti-NMDAR-E. In particular, teratomas associated with anti-NMDAR-E frequently contain neuroglial tissue and prominent immune-cell infiltrates, supporting a potential role of the tumor microenvironment in triggering or sustaining the autoimmune response. Higher anti-NMDAR antibody titers, rather than these histopathological features themselves, have been associated with poorer clinical outcomes [10,31,47,58]. Effective management requires the integration of neuropsychiatric and gynecological—and in more advanced cases, neurological, oncological, and surgical—perspectives [63].

This overall clinical constellation of symptoms, typically combining cognitive and behavioral alterations, such as psychosis or catatonia, with movements and speech abnormalities like orofacial dyskinesias creates a repeated pattern of the temporal, pathognomonic, and clinical trajectory of anti-NMDAR-E. The recognition of the above characteristics should strictly warrant prompt testing for antibodies against the GluN1 subunit of the NMDAR, especially in young individuals [5].

## 5. Diagnostic Findings

### 5.1. CSF Analysis

**Figure 3:**
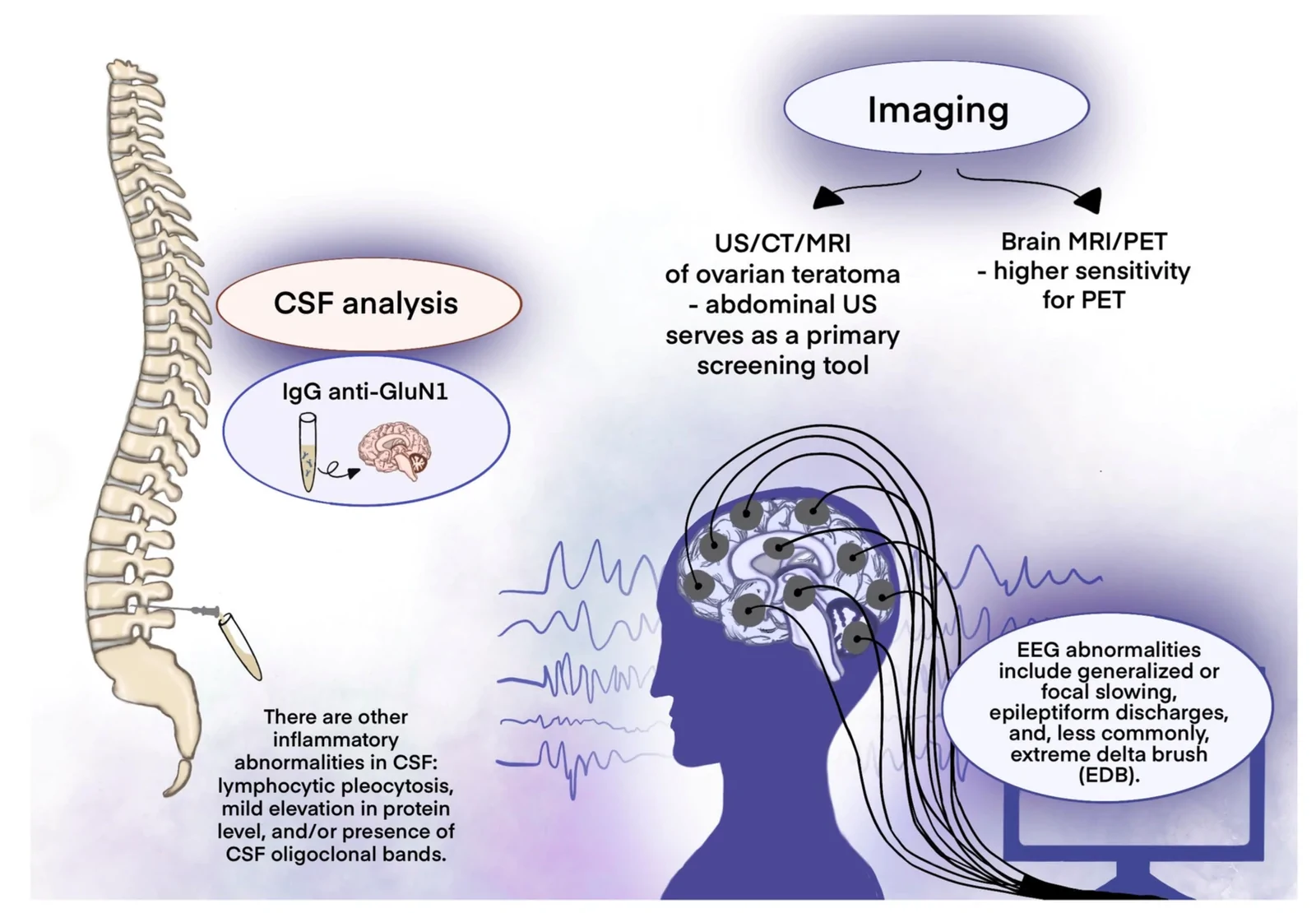
Diagnosis of anti-NMDAR-E in the CSF. Key diagnostic findings in screening for anti-NMDAR-E are the detection of anti-GluN1 IgG in CSF. Additional investigations include CSF analysis, EEG, brain imaging, and screening for and underlying ovarian teratoma. Based on Refs. [3,5,39,58,59,63,71,72,73,74,75]. Abbreviations: CSF, cerebrospinal fluid; IgG anti-GluN1, immunoglobulin G1 binding to subunit GluN1 of NMDA receptor; US, ultrasound; CT, computed tomography; MRI, magnetic resonance imaging; PET, positron emission tomography; EEG, electroencephalography.

Routine cerebrospinal fluid (CSF) and serum testing in patients with suspected anti-NMDAR-E is essential for establishing an accurate diagnostic assessment (Figure 3) [3]. The primary investigative objective is the detection of IgG antibodies that specifically bind to the GluN1 subunit of the NMDAR [3,59]. Although these specific IgG antibodies may be present in both CSF and serum, CSF analysis provides almost complete sensitivity and substantially greater diagnostic reliability, and therefore, the detection of IgG anti-GluN1 in CSF is considered a criterion for definitive diagnosis (Figure 3) [47,59,70]. Patients with confirmed disease may have corresponding negative serum results, which further underscores the superior diagnostic value of CSF testing [39,47,70].

Quantitative analyses also demonstrate that antibody titers in CSF tend to be higher in patients with an underlying ovarian teratoma as well as in those with poorer clinical outcomes [47]. In addition to IgG anti-GluN1 antibodies, CSF IgA NMDAR antibodies have been proposed as potential biomarkers of an associated ovarian teratoma. Desestret et al. found that CSF IgA NMDAR antibody positivity was significantly associated with the presence of ovarian teratoma, whereas no specific clinical features or clinical outcome were associated with IgA antibody positivity [40]. Moreover, longitudinal CSF testing demonstrated a decline in antibody titers during follow-up [47]. Importantly, relapse-associated changes in antibody titers were more frequently detected in CSF than in serum [47]. Beyond antibody detection, the CSF frequently exhibits nonspecific inflammatory abnormalities, most commonly lymphocytic pleocytosis, a mild elevation in protein level, and/or the presence of CSF oligoclonal bands [3,59,75]. Importantly, limitations of serum and CSF antibody testing include the possibility of rare early false-negative results, which may necessitate retesting when clinical suspicion persists [3,47].

Nevertheless, the gold standard for detecting pathogenic IgG in CSF is a cell-based assay (CBA) targeting antibodies against the GluN1 subunit of the NMDAR [47,76]. Both live- and fixed-cell CBAs are employed in clinical practice, although live CBAs are generally considered as the reference method [76]. Results may be corroborated by brain tissue immunohistochemistry minimizing false-positive and false-negative findings [47,70,77].

### 5.2. Imaging of Ovarian Teratoma

The first-line imaging modality in women undergoing evaluation for ovarian teratoma-associated anti-NMDAR-E is abdominal ultrasonography (US), which serves as a primary screening tool for tumor detection, though its variable sensitivity and limited ability to detect small or delayed-presenting lesions necessitate further imaging assessment (Figure 3) [63,72]. Transvaginal pelvic US provides more accuracy than a transabdominal approach [72]. Typically, a teratoma on ultrasonography manifests as adnexal mass, most commonly cystic in nature, containing an eccentric protuberance known as the Rokitansky protuberance, which may encompass a heterogenous mix of fat, calcifications, and other tissues such as hair or teeth [24]. However, when US findings are inconclusive or clinical suspicion persists despite negative results, pelvic magnetic resonance imaging (MRI) offers enhanced capability for detecting small, bilateral, or morphologically atypical teratomas that may be occult on US (Figure 3) [72]. It has been indicated that teratomas in anti-NMDAR-E tend to be smaller, contributing to a risk of being missed on routine US, MRI, or CT [58,78]. Contrast-enhanced computed tomography (CT) is particularly effective in demonstrating intratumoral fat and calcified components, whereas MRI provides superior soft-tissue resolution, with both techniques and combinations of multiple modalities facilitating the comprehensive evaluation of lesion features and precise identification (Figure 3) [71,79]. Nevertheless, a negative pelvic US or MRI/CT does not exclude the possibility of anti-NMDAR-E linked to an ovarian teratoma that was not identified on initial imaging [78,80,81,82]. In such cases, FDG-PET scanning has been reported to reveal hypermetabolic lesions consistent with teratoma; however, it is not routinely employed (Figure 3) [78].

### 5.3. Brain MRI and PET

Brain MRI may be used as a contributing diagnostic component but cannot be considered definitive, as, in many cases including patients with advanced disease, the imaging findings remain normal [3,83]. Although MRI of the brain can be valuable in differentiating anti-NMDAR-E from similar neurological disorders, its overall diagnostic reliability remains limited, as patterns are frequently nonspecific or even entirely unremarkable [63]. The prevalence of brain MRI abnormalities in anti-NMDAR-E varies substantially across studies, ranging from approximately 11% to 83% in different cohorts, as reported in recent analyses and systematic reviews [84]. The classic imaging profile of anti-NMDAR-E typically consists of T2/FLAIR hyperintensities with a predilection for unilateral or bilateral hippocampal involvement, whereas abnormalities within the infratentorial brain parenchyma and spinal cord are rare. Sporadically, leptomeningeal enhancement may also be detected [85,86].

It has been indicated that functional imaging with brain 18F-FDG PET demonstrates greater sensitivity than structural MRI, showing characteristic patterns of frontotemporal and striatal hypermetabolism with relative parietal–occipital hypometabolism [73].

### 5.4. EEG

In most patients, EEG demonstrates abnormal findings [5,39,87]. EEG patterns of anti-NMDAR-E may present as focal or diffuse slow or disorganized activity or evidence of epileptiform activity (Figure 3) [52,59]. Extreme delta brush (EDB) is recognized as a distinctive electroencephalographic pattern specifically linked to anti-NMDAR-E. However, despite its relative specificity, it is not a reliably sensitive feature (Figure 3) [74,88]. Jeannin-Mayer et al. analyzed several additional EEG abnormalities, including excessive beta activity (EBA), slow waves, EDB, GRDA (Generalized Rhythmic Delta Activity), spikes, sharp waves, and seizure activity. The study demonstrated a temporal sequence of EEG changes involving EBA, EDB, and GRDA and their occurrence in 71%, 58%, and 50% of patients, respectively [89]. From a clinical and prognostic standpoint, EEG may serve as a more valuable tool than MRI, especially for patients exhibiting delta-range abnormalities or EDB, as those factors are associated with a higher likelihood of ICU admission and poorer outcomes [87,88].

### 5.5. Diagnosis

The diagnosis of probable anti-NMDAR-E, according to the framework proposed by Graus et al., is allowed when three key criteria are met. These include the following: First, there must be a rapid onset (less than 3 months) of at least four of the six proposed major groups of clinical symptoms, namely psychiatric/behavioral abnormalities or cognitive dysfunction, speech dysfunction (verbal reduction, mutism, pressured speech), seizures, movement disorders (dyskinesias, muscle rigidity/abnormal postures), decreased level of consciousness, autonomic dysfunction, or central hypoventilation. Second, there are relevant EEG and/or CSF abnormal findings. Third, there is the exclusion of alternative diagnosis through appropriate differential assessment (Figure 2) [59]. Alternatively, probable anti-NMDAR-E may also be diagnosed in patients exhibiting three symptoms of the major clinical groups in the presence of a systemic teratoma [59].

Anti-NMDAR-E is considered definite when at least one suggestive clinical symptom is accompanied by the detection of IgG antibodies against GluN1 detected in CSF after other possible causes have been ruled out [59].

## 6. Therapeutic Strategies

### 6.1. Acute-Phase Management and Intensive Care

Once anti-NMDAR-E is diagnosed, there is a need to further assess the complications in nervous, respiratory, and cardiovascular systems [90]. Especially, those with teratoma may present more severe neurological conditions, higher incidence of fever, and arrhythmia. They more often need mechanical ventilation [91].

Consequently, most patients are admitted to an intensive care unit [92]. Supportive measures include the management of seizures, agitation using antipsychotics, antiepileptics, continuous cardiovascular monitoring, and ventilatory assistance [93].

### 6.2. Surgical Removal of Ovarian Teratoma

Underlying tumors are considered a major cause of autoimmune encephalitis; thus, the removal of even small tumors should be a part of comprehensive treatment that may decrease the risk of relapse and improve long-term outcomes (Figure 2) [91].

The surgical approach depends on tumor histology. Mature teratomas are usually treated with simple excision, while immature teratomas are usually treated with unilateral salpingo-oophorectomy with staging. Bilateral resection is reserved for bilateral disease [91]. Iyengar et al. showed that aggressive ovarian resection does not confer improved one-year functional outcomes in comparison to more conservative approaches, such as function-sparing resection in patients with anti-NMDAR-E [92]. Early surgery provides the opportunity for better management of the disease. Dalmau et al. showed that patients who underwent tumor identification and resection within the initial four months following the onset of neurological symptoms demonstrated improved outcomes compared to the remaining patient cohort [70]. Resection lowers relapse risk by approximately 23% [91]. Additionally, relapses are less common in patients with ovarian teratoma undergoing surgical procedure compared with patients without ovarian teratoma [91,94].

### 6.3. First-Line Immunotherapy

The standard first-line regimen includes high-dose intravenous corticosteroids, intravenous immunoglobulin (IVIG), and plasma exchange (PLEX) (Figure 2) [63]. It was reported that patients receiving immunotherapy in combination with surgery showed faster neurological recovery and improved outcomes [95]. A full recovery may be achieved in more than 80% of women with mature teratoma and more than 70% of women with immature teratoma over a period of several months to years (Figure 2) [58]. Delayed tumor removal, which is defined as a time interval of one month or more between the onset of symptoms and surgery, or delayed initiation of immunotherapy for more than 4 weeks after symptom onset, is associated with poorer 1-year functional status (*p* = 0.012) [96,97]. The effectiveness of immunotherapy in patients with teratoma improves significantly after surgical removal, which is relatively ineffective before surgery [94]. These observations suggest that immunotherapy often remains an important component of treatment even after tumor resection. Management requires a multidisciplinary team approach.

### 6.4. Second-Line Immunotherapy

In cases where surgical removal cannot be performed or there is no significant clinical improvement with first-line therapies, second-line treatment includes rituximab (chimeric anti-CD20 antibody) or cyclophosphamide (Figure 2) [90]. In pediatric patients, second-line agents should be administered within 10–14 days of insufficient response [98,99]. Rituximab can be used alone or in combination with cyclophosphamide. A multicenter Australian cohort study of 67 adult patients evaluated whether a single course of rituximab reduces relapse risk and how long its protective effect lasts. Based on the results, the protective effect of the monoclonal antibody persisted for approximately 6 months after each treatment course (HR 0.05; 95% CI 0.00–0.48; *p* = 0.005) and was associated with an 89% (HR 0.11; 95% CI 0.02–0.70; *p* = 0.02) reduction in hazard of first relapse. Furthermore, when the initiation of immunotherapy was delayed for more than 30 days after symptom onset, the relapse risk was increased (HR 5.81; 95% CI 1.89–17.82; *p* = 0.002) [100]. Notably, the presence of teratoma diminished the response to immunotherapy [94]. This may be related to ectopic NMDAR expression and persistent infiltration by lymphocytes that produce GluN1-IgG in the teratoma tissue [36,94,101].

### 6.5. Third Line and Emerging Therapies

Approximately 20–25% of patients exhibit refractory disease, and first- and second-line therapy remains insufficient. Third-line treatment includes tocilizumab and bortezomib (Figure 2). Especially, tocilizumab, an anti-IL6-receptor monoclonal antibody, provides an option in patients resistant to first-line treatment or rituximab [96,102]. The effect of combined immunotherapy with tocilizumab (T)-SIRT—presence of teratoma, administration of steroid, IVIG, rituximab, and tocilizumab) suggested better outcomes, especially when completed within 1 month, than conventional regimens without tocilizumab [96]. However, more studies should be conducted to assess the efficacy of this regimen (Figure 2). Novel findings suggest that CD20 monoclonal antibodies, especially ofatumumab, may be an alternative to rituximab and cyclophosphamide for patients with anti-NMDAR-E [103,104,105]. Moreover, a novel humanized monovalent antibody, ART5803, selectively binds to the GluN1 N-terminal domain (NTD), which prevents pathogenic antibody binding without receptor internalization. As a result, synaptic function in preclinical models is restored [57]. The NCT07093333 trial will assess the safety, tolerability, and pharmacokinetics of this drug in adult participants with a confirmed diagnosis of anti-NMDAR-E or anti-NMDAR antibody-associated psychiatric disease. Rarely, adjuvant chemotherapy using etoposide and cisplatin combination provides complete neurological response [106,107]. Table 1 summarizes selected ongoing and emerging therapeutic approaches in NMDAR-E treatment, including ART5803, inebilizumab, and satralizumab.

### 6.6. Long-Term Outcomes, Complications, and Rehabilitation

Recovery from anti-NMDAR-E typically unfolds over ≥18 months [5]. Regression of abnormalities of the autonomic nervous system can be observed within a few weeks, followed gradually—months to years—by improvement of the remaining symptoms, especially noticeable after tumor removal [90]. After 36 months of disease onset, one-third had impairment in one or more cognitive function, and two-thirds scored below-average on at least one domain as compared with the normative population. Approximately 70% of patients with anti-NMDAR-E returned to their duties in school or work, and a quarter of them later returned at a lower level [110].

Although overall prognosis remains favorable, patients may experience long-term cognitive deficits and increased levels of neuropsychiatric symptoms, including post-acute depressiveness, anxiety, sleep problems, and increased fatigue during recovery. Memory and concentration difficulties are also commonly observed [111]. Moreover, younger age at onset is associated with worse long-term adaptive behavior despite no differences in neurologic disability [112].

Impairment of cognitive outcomes occurs more frequently in patients with greater severity of disease during the acute phase, delayed treatment, and longer acute phase of the disease [113]. The most common impairments were working memory, verbal episodic memory, and executive function because of the highest density of NMDAR distribution in the hippocampus and frontal lobes [114,115,116]. Most patients showed no or minimal affective symptoms of depression and anxiety at the first study visit [117]. Between 10 and 32% of patients experienced moderate or mild symptoms of depression. Anxiety was reported in 11.5–60% of patients with the intensity being clinically relevant, moderate, or mild [117,118,119].

Given these challenges, neuropsychological assessment, proper memory strategies, and rehabilitation should be implemented. Each patient requires a multidisciplinary care approach involving psychiatrists, neurologists, psychotherapists, neuropsychologists, and speech or occupational therapists to provide proper recovery [111]. Immunotherapy-related (SIR and SIRT) adverse events include pneumonia, urinary tract infection, neutropenia, lymphopenia, acute kidney injury, and eosinophilia [96]. They should be monitored and managed properly to improve the quality of the treatment.

### 6.7. Fertility and Pregnancy Considerations

Anti-NMDAR-E is one of the leading causes of non-infectious encephalitis in pregnancy, with ovarian teratoma present in approximately 35.9–43% of pregnancy-associated cases [120,121]. Maternal NMDAR antibodies, predominantly of the IgG1 subclass, may cross the placenta and be transferred to the fetus, with potentially harmful effects [122]. Furthermore, NMDAR-IgG antibodies can remain detectable even after clinical remission [36,123]. Thus, it is important to consider not only disease onset during pregnancy, but also cases in which both onset and recovery occurred prior to pregnancy [121]. Neurological symptoms in infants are likely due to a combination of the transient pathogenic effects of maternal antibodies and the effects of antiseizure medications (ASM), immunomodulatory therapies (IMT), and other drugs used during pregnancy [122]. Newborns of mothers with anti-NMDAR-E may present with both neurological and non-neurological perinatal symptoms, including movement abnormalities, impaired reflexes, respiratory distress, infections, hypoglycemia, and low birth weight [120]. However, most newborns are healthy at birth. Joubert et al. reported that 90% of analyzed cases were healthy with normal 5 min APGAR scores at birth despite exposure to NMDAR antibodies [122]. The APGAR score is a rapid clinical assessment performed at 1 and 5 min after birth to evaluate a newborn’s condition based on heart rate, respiration, muscle tone, reflexes, and skin color [124]. Furthermore, all children with available follow-up showed normal development [122]. Harris et al. found no strong evidence of adverse neonatal outcomes, although reporting was generally limited. Among fifty-three neonates, seven (13%) were clinically compromised, with low Apgar scores, respiratory insufficiency, or neurological abnormalities [121]. In most tested cases with adverse outcomes, neonatal NMDAR-IgG seropositivity was detected, supporting a potentially harmful transplacental transfer of maternal autoantibodies [120,121,122,125,126]. Ueda et al. also highlighted that the absence of maternal serum NMDAR antibodies was associated with favorable neonatal outcomes [127]. A review found no significant differences in maternal outcomes, with 36/51 (71%) of pregnancy-associated cases achieving full recovery or minimal disability compared to 918/1284 (72%) achieving a Modified Rankin Scale (mRS) score of 0–2. Maternal mortality rates were also comparable (4/51, 8% vs. 81/1284, 6%) [121,128]. Overall, the association between anti-NMDAR-E and pregnancy remains incompletely understood [129]. The literature is limited and still evolving; however, the management of pregnancy in anti-NMDAR-E requires an individualized approach to balance maternal and fetal risks related to disease activity, autoantibodies, and therapy [120,121].

Beyond pregnancy outcomes, fertility preservation is an important consideration in women of reproductive age with anti-NMDAR-E. Ovarian teratoma removal may impact the future fertility of the patient, especially when both ovaries are involved. However, fertility preservation is often achievable, and it has been reported in several case reports. Ovarian tissue cryopreservation may be an option when the clinical course of the disease is more severe. Nevertheless, the careful pathological evaluation of the contamination risk of preserved tissue with teratoma components is essential [130].

## 7. Prognosis

### 7.1. Functional Outcome Metrics (mRS, CASE)

The MRS is an established scale used to evaluate the level of daily functional impairment or dependence in patients affected by stroke or other neurological disorders (Table 2) [10,91]. It is commonly used for measuring outcome and treatment response during hospitalization and in the follow-up periods in patients with anti-NMDAR-E [91,131]. A good outcome is defined as a mRS score ranging from 0 to 2 points, in contrast to a mRS score ranging from 3 to 6 points, which are considered unfavorable outcomes. Clinical improvement is characterized by a reduction of ≥1 point in the mRS score relative to the previous visit. A poor response is identified when the mRS score shows no improvement, or the mRS score remains ≥4 for a duration of four weeks [91]. The Titulaer et al. study included 179 patients with ovarian teratoma associated with anti-NMDAR-E who were treated with first-line immunotherapy (most often, steroids and IVIG) and tumor removal if applicable. Clinical improvement within 4 weeks and a mRS of 0–2 within 24 months occurred in 103 patients. Those without early improvement could receive second-line immunotherapy, which proved beneficial: second-line treatment significantly increased the odds of a good outcome by approximately 170% in patients who failed first-line immunotherapy (odds ratio [OR] 2.69; CI 1.24–5.80; *p* = 0.012) [5].

The Clinical Assessment Scale in Autoimmune Encephalitis (CASE) is also an alternative tool that can be used to estimate outcomes more precisely. It consists of nine symptoms—seizure, memory dysfunction, psychiatric symptoms, consciousness, language problems, dyskinesia/dystonia, gait instability and ataxia, brainstem dysfunction, and weakness—which are scored from 0 to 3, resulting in a total score ranging from 0 to 27. An increased CASE score correlates with a poorer prognosis [96,132].

The CASE and mRS generally demonstrate a moderate correlation [133]. However, the CASE addresses key limitations of the mRS, particularly its limited sensitivity to non-motor symptoms in autoimmune encephalitis, including neuropsychiatric manifestations such as memory impairment and psychiatric dysfunction [134,135]. In teratoma-associated anti-NMDAR encephalitis, patients typically present with predominantly neuropsychiatric phenotypes, which is better captured by the CASE score, whereas the mRS primarily reflects global functional disability [10,63]. Accordingly, CASE demonstrates greater sensitivity to clinical changes, whereas differences between the two scales are most evident in severe disease and less pronounced in milder phenotypes. Therefore, CASE is useful for monitoring disease severity and treatment response. However, clinical use of the CASE remains limited, and construct validity is not yet fully established; therefore, further validation across subtypes and populations is required [133,134].

**Table 2:** Modified Rankin Scale used for functional outcome assessment in anti-NMDAR-E. Based on Ref. [136].

Modified Rankin Scale—mRS	
Grade 0	No neurological symptoms
Grade 1	Minor symptoms without significant impact; able to perform all usual activities
Grade 2	Mild disability; unable to do all previous activities; can manage own affairs independently
Grade 3	Moderate disability; needs some help; able to walk without assistance
Grade 4	Moderately severe disability; unable to walk without assistance
Grade 5	Severe disability; bedridden; incontinent; requires continuous care
Grade 6	Death

### 7.2. Other Factors

Early treatment, no need for admission to an ICU, and a low level of anti-NMDAR antibodies in CSF and in serum are associated with good outcome [5,97]. A longer time to treatment initiation is associated with significantly worse outcomes. In the multivariable model, Titulaer et al. presented that increasing (log-transformed) treatment delay resulted in lower odds of achieving a good outcome by approximately 38% (OR 0.62 CI 0.50–0.76; *p* < 0.0001). Moreover, ICU admission is a strong predictor of poor outcome, reducing the odds of achieving a favorable outcome by 88% (OR 0.12 CI 0.06–0.22; *p* < 0.0001) [5].

It is also worth mentioning that there are two symptoms that occur significantly more frequently in patients with tumors—central hypoventilation and decreased consciousness [91,131]. If these symptoms appear in encephalitis, they may be associated with a higher risk of poor functional status: 39% versus 18% without central hypoventilation and 31% versus 18% without reduced consciousness [97].

### 7.3. Age

Age younger than 2 years and older than 65 years at the initiation of the disease is associated with increased odds of poor outcome (3.6-fold and 3.8-fold), while adolescents (12–19 years) had 2.6-fold increased odds for good outcome compared to patients aged between 20 and 65 years at the onset of anti-NMDAR-E [128]. Moreover, among female patients below the age of 45, poorer prognosis tended to occur in patients with older age [137]. Younger age seems to be a risk factor for the occurrence of immature teratomas, with a median age of 22 years old (12–38 years), compared to MT, with a median age of 25 (15–45) [10,30].

### 7.4. Relapse of Encephalitis

The definition of a relapse of encephalitis is the worsening of previous symptoms with the increase of ≥1 in mRS or the onset of new ones after at least two months of improvement or stability [10]. Relapses occur more often in non-tumor patients than in those diagnosed with tumors. The paraneoplastic origin of the disease is associated with a lower frequency of relapses. It decreases the risk by 68% (HR 0.32; CI 0.12–0.87; *p* = 0.02). Overall, the relapse rate of encephalitis in patients with ovarian teratoma was approximately 15% and 24–33.3% without tumor [91,138]. On the contrary, Titulaer et al. reported a mean relapse frequency of 12%. The difference between studies could be caused by different diagnostic criteria and earlier onset of treatment. The mean time to relapse in patients with ovarian teratoma is longer than without teratoma (71 ± 4.463 vs. 43.001 ± 3.401).

Relapses of anti-NMDAR-E are less severe than the first episode. The number of symptoms during relapses is significantly lower than in the first episode of the disease. According to the study by Ciano-Persen et al., approximately 55% of patients have only one symptom and 33% have two symptoms. In the case of the first episode of the disease, approximately 89% of patients have three or more symptoms. Moreover, the maximal mRS recorded at the first episode was higher than that at relapse (median 5, range 3–5, IQR 1 vs. median 3, range 0–6, IQR 1; *p* = 0.0001) [139].

In contrast to the positive influence on predictive functional outcome at 12 months, adolescent age (12–19 years) is associated with a higher risk of relapse (OR 2.18; CI 1.18–4.15; *p* = 0.01) [128]. These findings underscore the critical influence of early diagnosis, particularly at a young age, and highlight the importance of the prompt initiation of therapy to minimize the risk of relapse and improve long-term outcomes.

## 8. Future Perspectives

Despite major advances in understanding the pathophysiology of anti-NMDAR-E, many aspects of its pathophysiology, early diagnosis, and long-term management remain incompletely resolved. Current therapeutic approaches, including tumor resection and first-line immunotherapy, are effective for most patients, yet a significant number of them experience relapses or cognitive complications. Therefore, ongoing research is focused on identifying biomarkers of disease activity, refined animal models, and exploring novel therapeutic targets aimed at preventing or reversing antibody-mediated neuronal dysfunction.

Lao et al. created a mouse model of anti-NMDAR-E to study the process underlying BBB damage. They demonstrated that the activation of the p38 MAPK signaling pathway in brain endothelial cells resulted in increased vascular permeability, neuronal inflammation, and the loss of tight-junction proteins, such as occludin, ZO-1, and claudin-5. The treatment with selective p38 MAPK inhibitor SB203580 alleviated neurological symptoms, reduced leakage, and restored junctional integrity. These results suggest that p38 MAPK activation drives BBB disruption, suggesting that its inhibition could be a potential therapeutic approach in anti-NMDAR-E [140]. Building on these findings, Li et al. showed that peripheral immune activation also contributes to BBB dysfunction. Circulating monocytes from patients with anti-NMDAR-E exhibited elevated levels of interferon regulatory factor 7 (IRF7) expression and type I interferon signaling, leading to the release of pro-inflammatory cytokines and endothelial dysfunction promoting antibody entry into the CNS [141]. Together, these studies highlight both central and peripheral mechanisms contributing to barrier impairment and uncover signaling pathways that may be targeted to reduce CNS inflammation in anti-NMDAR-E.

Experimental models have provided important insights into the downstream effects of teratoma-induced autoimmunity. Ding et al. was the first to establish an active immunization mouse model of anti-NMDAR-E by repeatedly injecting C57BL/6 mice with a peptide corresponding to amino acids 356–385 of the GluN1 subunit’s amino-terminal domain. As a result, the immunized mice produced specific anti-GluN1 antibodies, which were detectable in serum and CSF. The mice developed hippocampal inflammation, loss of surface receptor clusters, impaired long-term potentiation (LTP), and memory deficits—closely mirroring what is observed in humans. This model demonstrated that antibody production alone is sufficient to reproduce the synaptic and behavioral consequences of anti-NMDAR-E [142]. Based on their work, Yang et al. examined the role of the NLRP3 inflammasome, a major innate immune signaling complex that increases neuroinflammation by activating caspase-1 and releasing IL-1β and IL-18, using a similar immunization strategy. Affected mice showed elevated NLRP3 expression in the hippocampus and microglial activation. The pharmacological inhibition of this pathway with MCC950 led to attenuated cytokine release, decreased neuronal injury, restored synaptic plasticity, and markedly improved learning and memory [143]. Together, these studies show that adaptive and innate immune responses act jointly to maintain neuroinflammation, providing a foundation for developing more targeted therapeutic strategies.

Maudes et al., in their 2025 study, developed an active immunization mouse model by injecting mice with GluN1 356–385, the same peptide as Ding et al. Similar to Ding’s results, this immunization led to the production of anti-GluN1 antibodies, reduced synaptic NMDAR density, hippocampal inflammation, and microglial activation. This mirrors the human disease phenotype. The affected mice showed memory impairment, abnormal movements, and behavioral alterations, such as anxiety and depressive-like symptoms. Active neuroinflammation was confirmed by histopathological analysis, which detected plasma cells and B-cell infiltrates and NMDAR-IgG immune complexes within microglial endosomes. The majority of electrophysiological and behavioral defects were successfully corrected by treatment with anti-CD20 antibodies and a positive allosteric NMDAR modulator (SGE-301), and combination therapy prevented relapse following B-cell repopulation [144]. Overall, this model connects mechanistic and therapeutic research, providing a valuable platform for testing targeted interventions in anti-NMDAR-E.

Among novel therapeutic strategies, the study by Kanno et al. is particularly noteworthy for introducing an antigen-specific, non-immunosuppressive strategy. ART5803, a humanized monovalent antibody, was developed that binds to the GluN1 amino-terminal domain, competitively blocking pathogenic antibody binding without inducing receptor internalization. Moreover, ART5803 reversed behavioral deficits, prevented NMDAR loss, and restored function in neuronal cultures and marmoset model [57].

In a mouse model of anti-NMDAR-E, Kong et al. showed that the mTOR inhibitor Rapamycin reduces oxidative stress and mitochondrial dysfunction. Rapamycin enhanced neuronal survival and cognitive function by restoring autophagy and mitochondrial homeostasis, indicating that cellular metabolism regulation may be a promising adjunctive strategy to protect against antibody-mediated neuronal injury [145].

Despite substantial advances in understanding ovarian teratoma-associated anti-NMDAR-E, the available evidence remains limited. The majority of the current knowledge comes from case reports, retrospective cohort studies, and observational studies, which may restrict the generalizability of reported findings and introduce selection bias. In addition, the rarity of the disease and heterogeneity of study populations complicate direct comparisons across studies. To strengthen the current evidence base and improve diagnostic and treatment approaches, more prospective multicenter investigations are required [5,146].

## 9. Conclusions

Ovarian teratomas associated with anti-NMDAR-E typically consist of abnormal neuroglial tissue expressing the GluN1 subunit, which may trigger a pathogenic autoimmune response. However, the underlying mechanisms for the loss of immune tolerance remain incompletely understood and require further investigation.

The clinical course of anti-NMDAR-E is challenging due to its nonspecific prodromal flu-like symptoms and rapid onset of psychiatric manifestations, such as disorganized behavior, agitation, and anxiety, which evolve into neurological symptoms and autonomic dysregulation. Consequently, anti-NMDAR-E may be misdiagnosed as a primary psychiatric disorder, resulting in delays in appropriate treatment and poorer clinical outcomes.

Surgical removal of ovarian teratoma combined with first-line immunotherapy (high-dose corticosteroids, IVIG, and plasma exchange) is associated with favorable long-term outcomes in the majority of patients when treatment is initiated early. Additional immunotherapeutic strategies may be required in refractory or relapsing cases.

Despite substantial advances in understanding ovarian teratoma-associated anti-NMDAR-E, important knowledge gaps remain. The mechanisms responsible for the loss of immune tolerance, factors influencing disease susceptibility, and determinants of treatment response require further investigation. Future research should prioritize the identification of reliable biomarkers, validation of emerging therapeutic strategies, and the development of prospective multicenter studies to strengthen the current evidence base. Continued progress will require close collaboration between gynecologists, neurologists, immunologists, and psychiatrists.

## Figures and Tables

**Table 1:** Ongoing and emerging therapeutic clinical trials in anti-NMDAR-E.

Investigational Agent	Target/Mechanism	Clinical Development	Potential Relevance	Study Objective	Reference
ART5803	Humanized monovalent antibody targeting the GluN1 N-terminal domain	Phase 2a (NCT07093333)	Selectively blocks pathogenic anti-NMDAR antibody binding to the GluN1 N-terminal domain without inducing receptor internalization, thereby preserving synaptic function	Evaluation of safety, tolerability, PK, and preliminary efficacy in anti-NMDAR-E	[57]
Inebilizumab	Humanized anti-CD19 monoclonal antibody	Phase 2b (ExTINGUISH; NCT04372615)	Depletion of B-cells and plasmablasts involved in autoantibody production	Evaluation of safety and efficacy in patients with acute NMDAR-E who experience moderate-to-severe disability after standard first-line immunotherapies	[108]
Satralizumab	Humanized monoclonal antibody targeting the IL-6 receptor	Phase 3 (NCT05503264)	Targeting IL-6-mediated neuroinflammation, persistent immune activation, and blood–brain barrier disruption in autoimmune encephalitis	Evaluation of efficacy, safety, PK, and pharmacodynamics in patients with anti-NMDAR-E or anti-LGI1 autoimmune encephalitis	[109]

Abbreviations: anti-NMDAR-E—anti-N-methyl-D-aspartate receptor encephalitis; NMDAR—N-methyl-D-aspartate receptor; GluN1—glutamate ionotropic receptor NMDA type subunit 1; IL-6—interleukin-6; CD19—cluster of differentiation 19; PK—pharmacokinetics; NCT—National Clinical Trial identifier.

## Data Availability

No new data were created or analyzed in this study. Data sharing is not applicable to this article.

## Abbreviations

AI	Artificial intelligence
APC	Antigen-presenting cell
ART5803	Humanized monovalent monoclonal antibody targeting the GluN1 N-terminal domain
BAFF	B-cell-activating factor
BBB	Blood–brain barrier
CASE	Clinical Assessment Scale in Autoimmune Encephalitis
CBA	Cell-based assay
CNS	Central nervous system
CSF	Cerebrospinal fluid
CT	Computed tomography
CXCL13	C-X-C motif chemokine ligand 13
EBA	Excessive beta activity
EDB	Extreme delta brush
EEG	Electroencephalography
ICU	Intensive care unit
IgA	Immunoglobulin A
IgG	Immunoglobulin G
IL-6	Interleukin-6
IL-17	Interleukin-17
IL-18	Interleukin-18
IL-21	Interleukin-21
IRF7	Interferon regulatory factor 7
IVIG	Intravenous immunoglobulin
LTP	Long-term potentiation
MCT	Mature cystic teratoma
MMP-9	Matrix metalloproteinase-9
MRI	Magnetic resonance imaging
MT	Mature teratoma
NLRP3	NOD-like receptor family pyrin domain-containing 3
NMDAR	N-methyl-D-aspartate receptor
Anti-NMDAR-E	Anti-NMDA receptor encephalitis
NR1	Former name of the GluN1 subunit of the NMDA receptor
NTD	N-terminal domain
OGCT	Ovarian germ cell tumor
PET	Positron emission tomography
PLEX	Plasma exchange
SB203580	p38 MAPK inhibitor
SGE-301	Positive allosteric modulator of NMDAR used in experimental models
SIR	Steroid and IVIG with rituximab regimen
SIRT	Steroid, IVIG, and rituximab regimen
T-SIRT	Teratoma removal with steroid, IVIG, rituximab, and tocilizumab regimen
TLS	Tertiary lymphoid structure
TNF-α	Tumor necrosis factor alpha
US	Ultrasound
mRS	Modified Rankin Scale
